# Apoptosis induced by acrylamide is suppressed in a 21.5% fat diet through caspase-3-independent pathway in mice testis

**DOI:** 10.1080/15376510802499048

**Published:** 2009-06-30

**Authors:** Xichun Zhang, Fahe Chen, Zhiyong Huang

**Affiliations:** Bio-tech Engineering College, Jimei University, Xiamen, Fujian, PR China

**Keywords:** Acrylamide, High-fat, Testis, Apoptosis, Caspase-3

## Abstract

This study investigates the simultaneous effect of acrylamide (ACR) and high-fat-intake on the apoptosis in testis cells, and also the expression and activity of caspase-3. Seventy-two male Kunming mice were divided into two blocks and fed with a high-fat diet (crude fat 21.5%) or basic diet (crude fat 4.4%), respectively; and animals in each diet block were exposed to ACR at the dose of 20 mg/kgbw•d or 40 mg/kgbw•d as ACR treated groups or the normal saline as control. Germ cells prepared from testis were stained with Hoechst dye 33258 and paraffin wax sections from testis were suffered to a TUNEL process. Expression of caspase-3 on protein level was investigated using an immunohistochemical analysis assay. The supernatant of unilateral testes were subjected to a Caspase-3 activity kit to determine the activity of Caspase-3 in testis. The concentration of ACR and glycidamide(GA), *epox-ide of ACR*, in plasma and testis were detected by LC-ES/MS/MS analysis. Results based on the morphological changes, percentage of apoptotic cells, and integrated optical density (IOD) of positive amethyst staining which indicates the apoptotic DNA fragmentation, show that apoptosis was induced by acrylamide only; however, acr-ylamide-induced apoptosis was weakened by high-fat-intake. The protein expression and activity of Caspase-3 were not induced by ACR or high-fat-intake. Moreover, no significant differences of ACR and GA concentration were found between the high-fat and basic diet groups after exposure of ACR. Results indicate that high-fat-intake reverses the effects on apoptosis induced by ACR; and more possibly, apoptosis is induced by a caspase-3-independent mechanism.

## Introduction

In 2002, researchers demonstrated relatively high level of acrylamide (ACR) in foods cooked at high temperature, especially in carbohydrate-rich foods (Tareke et al. 2002). These widely publicized findings stimulated worldwide studies on determining ACR level in food and on the nature of the ACR because the exposure of humans to ACR can come from both external sources and the diet.

ACR is reported, at a dose of 7–14 mg/kg, to reduce fertility rates, increase resorptions of fetuses, reduce litter size in pregnant females, and cause the formation of abnormal sperm and decrease sperm count in males (Sakamoto and Hashimoto 1986; Chapin et al. 1995). ACR-induced histopathological lesions, such as formation of multinucleated giant cells and vacuolation, and numerous apoptotic cells were observed in seminiferous tubules. Serum testosterone level and Leydig cell viability were also decreased dose-dependently, which resulted in decreased spermatogenesis (Yang et al. 2005). Although not active in the in vitro Ames test, ACR does elicit mutagenic effects in stem cell spermatogonia (Dearfield et al. 1995). ACR is also reported to induce dominant lethal mutations in spermatids (clastogenic or chromosome damaging effects) of mice and rats and is thus considered to be a mammalian germ cell mutagen (Shelby et al. 1987; Adler et al. 2002).

The molecular mechanisms of reproductive toxicity could be the result of alkylation of SH groups in the sperm nucleus and tail, depletion of GSH, or DNA damage in the testis (Dearfield et al. 1995). Moreover, the ACR induces chromatin adducts in germ cells of male mice, which indicates that ACR can reach sperm cell nuclei (Holland et al. 1999). A cytometer-based dose-response micronucleus assay shows that very low doses of ACR can damage chromosomes of mouse spermatids and the linearity of the dose-response suggests that ACR is DNA-reactive clastogens and represent a health risk to mouse spermatids (Adler et al. 2000).

Apoptosis, a selective process of physiological cell deletion that regulates the balance between cell proliferation and cell death, is induced by DNA damage or oxidative stress. Programmed germ cell death plays an indispensable role during fetal, neonatal, postnatal, and adult spermatogenesis (Mori et al. 1997; Wang et al. 1998; Sinha Hikim and Swerdloff 1999). During the apoptotic process, caspases are considered as a central player for the apoptotic process and cascade of proteolytic cleavage event. The Caspase-dependent process is associated with two pathways of apoptosis, a death receptor-dependent and a mitochondria-dependent pathway (Debatin 2004). Death receptor activation pathway is mediated with a death-inducing signaling complex, which is made of a Fas-associated death domain and a procaspase-8, activating Caspase-8 (Kim et al. 2002). Caspase-8 directly activates Caspase-3, leading to apoptosis. The mitochondria-dependent pathway involves cleavage of proapoptotic proteins by Caspase-8 releasing cytochrome C in the mitochondria. Released cytochrome C activates Caspase-3 and Caspase-9, inducing the apoptosis with morphological, biochemical, and mitochondrial changes (Li et al. 2003). Caspase-3, in particular, is associated with the execution of apoptosis and induces the large number of morphological changes characteristic of cells undergoing apoptosis (Shi 2002).

Although studies on ACR on reproductive toxicity have been carried out, the role of apoptosis in this process is still unclear. Moreover, high-fat-intake, which is common in diet and always exposes to humans together with ACR through lots of food such as fried potato, may interacts with ACR on reproductive toxicity induced by ACR. Epidemiological studies and laboratory animal model assays suggest that a high intake of dietary fat promotes mammary carcinogenesis. For an animal model, both the high-fat corn oil diet and high-fat mixed-lipid diet promote 7,12-dimethylbenz[a]anthracene-induced mammary carcinogenesis when compared to a low-fat corn oil diet (Kuno et al. 2003).

In the present study, we investigate the combining effect of ACR and high-fat-intake on the apoptosis in testis cells of Kunming mice. And then we investigate the expression and activity of caspase-3.

## Materials and methods

### Animal model and treatment

Kunmingmouse is an outbred stock derived from Swiss albino mice with a high heterogeneity of genes (Sun 2001) and is widely employed in studies on neuroscience (Yu et al. 1997), immunology (Feng et al. 2002), genetics (Bu et al. 2002), and pharmacology (Yang et al. 2002) in Chinese laboratories. Seventy-two male Kunming mice (Animal Center of Xiamen University, China), weighing 20–30 g, were housed in a SPF (specified-pathogens free) animal room maintained at constant temperature (22 ± 2°C) and relative humidity (40–60%) with a 12 hour light/dark cycle. Animals were assigned to treatment groups by stratified random sampling (12 mice per group). The mice were given two kinds of diet with different fat content. The basic diet contains 4.4% fat according to the requirement of mice; The high-fat diet, in which crude fat was 21.5%, includes 57.5% basic feed, 10% lard, 20% milk powder, 10% yolk powder, 2% cholesterol, and 0.5% bile pigment. Mice in two different diet groups were exposed to ACR at the dose of 20 mg/kgbw•d or 40 mg/kgbw•d and the normal saline that was used as control.

Normal saline in which ACR dissolved and also the normal saline (control) were intraperitoneally injected at a dose of 10 ml/kgbw (five times per week for 4 weeks).

Blood was collected via cardiac puncture using heparinized syringes and was immediately processed to separate the plasma for concentration measurements of ACR and GA. Testes were removed and immediately frozen using liquid nitrogen and stored at −80°C for spermatogenic cell preparation and caspase-3 activity measurements at a later time; or fixed after perfusion for histochemistry measurement.

### LC-ES/MS/MS analysis of ACR and GA in plasma and testis

Blood and testis were taken from three mice per acrylamide-treated group. Analyses of ACR and GA in plasma and testis were performed using a high-throughput LC-ES/MS/MS method as previously described (Twaddle et al. 2004). Briefly, labeled internal standards (^13^C_3_-ACR and ^13^C_3_-GA) were added to each thawed plasma sample or testis supernatant sample (10–100 ml); then samples were purified using solid phase extraction in 96-well plates and analyzed using LC-ES/MS/MS in the multiple reaction monitoring mode for ^13^C-labeled and unlabeled ACR and GA transitions.

### Spermatogenic cell preparations and fluorescence microscopy

Mixed germ cell preparations were obtained by enzymatic digestion. Briefly, testes were detunicated, digested in 0.5 mg/ml collagenase (Sigma, St. Louis, MO) in Krebs-Ringer bicarbonate (KRB) media at 32°C for 20 minutes, then digested with 0.5 mg/ml of trypsin (Sigma) and 1 μg/ml DNaseI (USB, Cleveland, OH) in KRB at 32°C for 13 minutes; germ cells were subsequently released by pipetting for 3 minutes. The germ cell suspension was filtered through a Nitex mesh and washed three times in 0.5% bovine serum albumin (BSA; Sigma) in KRB. After the final wash, cells were counted and resuspended at a concentration of 2.5 × 10^6^ cells/ml in KRB media; the cells were then centrifuged at 5000 rpm for 5 minutes. Then cells were fixed in 4% paraformaldehyde for 5 minutes. The cells were then stained with Hoechst dye 33258 (8 μg/ml) for 5 minutes, washed twice with phosphate buffered saline (PBS), and mounted. Nuclei were observed using an Axiovert 100 inverted fluorescence microscope.

### Histochemistry assay

Animals were anesthetized with ether, and then perfused with the normal saline through the vessel at 37°C till the tes-tes turn white. To keep the tissue shape from changing before embedding, perfusion was carried out with phosphate buffer (pH 7.4) containing 4% polyformaldehyde through artery at 4°C for 10 min. Then, testes were taken off on ice, immersed into 4% polyformaldehyde for 24 hours, dehydrated in 50–100% ethanol, cleared in xylene, and embedded in paraffin wax. The central cross-cut serial sections with a thickness of 4 μm were obtained for every 3 mm of the tissue.

A TUNEL kit (Wako, Osaka, Japan) was used for DNA fragmentation analysis according to the manufacturer's instructions. Briefly, the tissue sections were deparaffinized, rehydrated and treated with protein digestion buffer for 8 minutes at 37°C, followed by incorporation of biotin-deoxyuridine triphosphate in the presence of working strength deoxynucleotidyl transferase (TdT) for 10 minutes at 37°C. After blocking the endogenous peroxidase (POD) activity by incubation with 3% hydrogen peroxide (H_2_O_2_) in PBS buffer for 5 minutes at room temperature, the tissue samples were incubated with POD-conjugated antibody for 10 minutes at 37°C, developed with dimethylaminoazobenzene (DAB) for 5 minutes at room temperature, and finally counterstained with methyl-green and examined under a MOTIC Biological Microscope (Micro-optic Industrial Group, Xiamen, PRC). Cells with amethyst staining, indicative of DNA fragmentation, were considered to indicate apoptosis and the integrated optical density (IOD) of positive amethyst staining was analyzed with Images Pro Plus 5.0.1 (Micro-optic Industrial Group, Xiamen, PRC) to indicate the expression level of protein.

Tissue sections of sham-injected mice pretreated with DNase I prior to incubation with TdT were used as a positive control, while tissue sections pre-treated with TdT substrate solution without TdT served as a negative control in TUNEL assay.

Immunohistochemical analysis (IMC) was used to investigate the apoptotic caspase-dependent pathway. Caspase-3 plays an essential role as an executor in apoptosis (Porter and Janicke 1999). Therefore, we examined whether ACR or high-fat-intake activates caspase-3 during the induction of apoptosis in germ cells. Sections were deparaffinized in xylene and hydrated in gradient ethanol. Endogenous peroxidase was blocked with 3% H_2_O_2_ in 70% methanol. Then sections were washed for 10 minutes in PBS (pH7.3), and non-specific protein-binding sites were blocked with 5% normal goat serum to reduce background staining. Next, immunolocalization of Caspase-3 were processed using a monoclonal antibody against Caspase-3 (1:600 dilution; Santa Cruz Biotechnology, Santa Cruz, CA) as the primary antibodies. Sections were then incubated with the biotinylated secondary antibody, rabbit anti-mouse IgG, and then strep avidin-biotin complex (SABC) agent (Boster biotechnology, Wuhan, PRC) for 20 minutes at 37°C. After a 5-minute wash, the sections were treated with liquid diaminobenzidine (Boster biotechnology, Wuhan, PRC) and counterstained with hematoxylin. The stained sections were photographed using a MOTIC Biological Microscope (Micro-optic Industrial Group, Xiamen, PRC). IOD of positive immunostain was analyzed with Images Pro Plus 5.0.1(Micro-optic Industrial Group, Xiamen, PRC).

### Caspase-3 activity

Caspase-3 activity was measured using testis supernatants with the Caspase-Glo Assay System (Promega, Madison, WI) according to the manufacturer's instructions. Briefly, 50 μl of the Caspase-Glo buffer was added to 50 μl of tissue supernatants in a 96-well plate and incubated at room temperature for 60 minutes. Luminescence was measured using a Biotek synergy microplate reader (Biotek, Winooski, VT). Background activity levels, based on measurements in homogenization buffer only, were measured and subtracted from values in the testis tissue supernatants. All values were normalized to total protein content. Standard curves were used to determine the linearity of the responses.

### Statistical analysis

All data were expressed as mean ± SD. Means of different groups were compared with Repeated Measured Two Factors ANOVA (*F*-test). Multi-comparison was taken by LSD or SNK (*Q*-test).

## Results

### Concentration of ACR and GA in plasma and testis

Concentration of ACR and GA in the plasma and testis are presented as mean ± SD of three animals. No significant differences of ACR and GA concentration were found between the high-fat and basic diet groups after exposure of ACR (Table 1).

**Table 1:** Acrylamide and glycidamide concentration in the plasma and testis (μg/mg).

			ACR		GA	
				
		*n*	Plasma	Testis	Plasma	Testis
NM	ACR 20 mg/kgbw•d	3	5.5 ± 2.2	4.8 ± 3.1	4.4 ± 0.4	2.6 ± 0.1
	ACR 40 mg/kgbw•d	3	13.4 ± 3.2	9.2 ± 2.4	8.9 ± 0.9	7.6 ± 1.1
HF	ACR 20 mg/kgbw•d	3	6.9 ± 2.3	6.5 ± 4.0	3.5 ± 0.2	2.3 ± 0.3
	ACR 40 mg/kgbw•d	3	14.2 ± 2.4	8.6 ± 1.8	7.4 ± 2.0	7.4 ± 0.5

*n* = number of mice used in experience, NM = normal diet block, HF = high-fat diet block. Acrylamide and glycidamide concentration in the plasma and testis are presented as mean ± SD of three animals. No significant differences of acrylamide and glycidamide concentration were found between the high-fat and basic diet groups after exposure of acrylamide.

### Apoptosis induced by ACR and high-fat-intake

**Figure 1:**
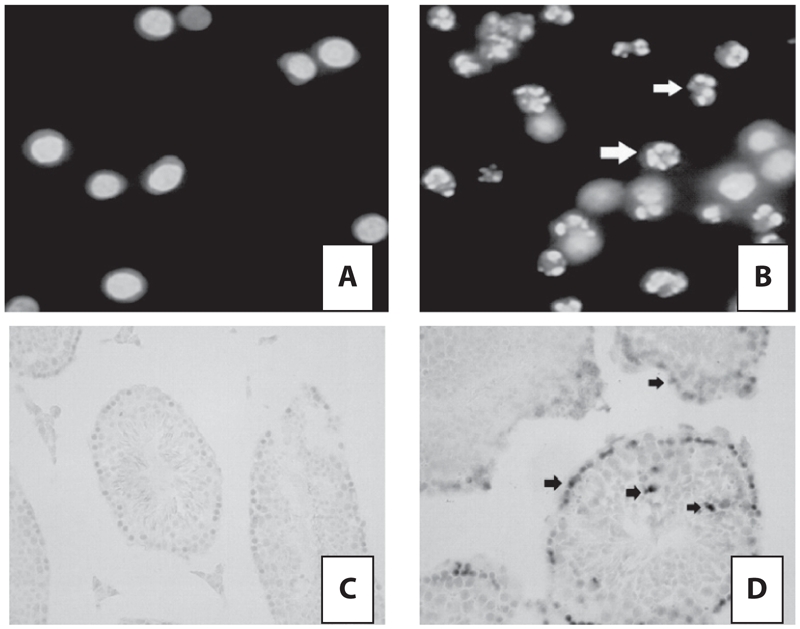
Apoptosis induced by acrylamide or high-fat. Apoptotic body formation (open arrow) was observed under the microscope in acryla-mide-treated mice testis (B), but not those treated with acrylamide and high-fat together (A). And, as shown, the apoptosis cells with amethyst staining (arrow) were observed in acrylamide-treated mice testis (D), but not those treated with acrylamide and high-fat together (C).

Apoptosis was first examined by observation of morphological changes of cells. Three testes from mice per group were used to prepare the spermatogenic cells and the cells fixed in sections were subjected to a fluorescence observation. The number of cells taking the formation of apoptotic body were counted. The total cell number was not determined by dividing cell types such as testicular germ cells and sertoli cells because it was difficult to determine the difference of cell types which has no significant differences in nuclear size (Russell et al. 2002). Thus, the total number of cells reflected all cell types of seminiferous tubules. Apoptotic change like formation of apoptotic body was observed under the microscope in ACR-treated mice testis; the percentage of apoptotic cells in ACR-treated mice testis was significantly higher than control (ACR-non-exposed group with normal diet). Whereas, no observed apoptosis and significant changes of percentage of apoptotic cell were detected out in any mice testis treated with ACR and high-fat together (Fig. 1A and 1B, Table 2).

**Table 2:** Apoptosis induced by acrylamide and high-fat intake.

		Fluorescence microscopy		TUNEL assay		
			
		*n*	Apoptotic cell %	*n*	Staining cells %	IOD of amethyst staining
NM	ACR 0 mg/kgbw•d	3	2 ± 0.5	6	23 ± 2	198.34 ± 56.83
	ACR 20 mg/kgbw•d	3	15 ± 2^a^	6	158 ± 21^a^	2007.03 ± 1025.61^a^
	ACR 40 mg/kgbw•d	3	16 ± 3^a^	6	169 ± 23^a^	4325.37 ± 208.13^a^
HF	ACR 0 mg/kgbw•d	3	3 ± 0.2	6	26 ± 5	112.23 ± 98.51
	ACR 20 mg/kgbw•d	3	4 ± 0.7	6	28 ± 4	158.41 ± 65.96
	ACR 40 mg/kgbw•d	3	3 ± 0.1	6	31 ± 5	162.51 ± 58.27

*n* = number of mice used in experience, NM = normal diet block, HF = high-fat diet block.

a *p* < 0.05 (compared to NM 0 mg/kgbw•d).

ACR significantly increased the apoptosis which was indicated by the apoptotic cell number of total spermatogenic cells and the IOD of apoptotic cells (amethyst staining) per animal in acrylamide-treated mice testes. However, no significant change occurred on the apoptotic cell number of total spermatogenic cells and the IOD of apoptotic cells in mice testes treated with acrylamide and high-fat together compared to control.

Six testes from mice of a group, five sections of a mouse testes, were carried a TUNEL process. As shown in Fig. 1C and 1D and Table 2, the apoptosis cells with amethyst staining were observed in ACR-treated mice testis rather than those treated with ACR and high-fat together. The IOD of apoptotic cells (amethyst staining) significantly increased in ACR-treated mice testis rather than that treated with ACR and high-fat together (Table 2). Results above indicated that apoptosis was induced by ACR only rather than ACR and high-fat diet together.

Taken together, data demonstrate that high-fat-intake weakens the up-regulation of ACR-induced apoptosis in mouse testes.

### Expression and activity of Caspase-3

In order to investigate the apoptotic caspase-dependent pathway, we used an immunohistochemical analysis assay to determine the expression of caspase-3 on protein level. Five sections from every testis of six mice of a group were carried out for the immunohistochemical analysis. The IOD was used to indicate the expression level of protein and the results are shown in Table 3. ACR (20 mg/kgbw•d and 40 mg/kgbw•d) did not significantly increased the Caspase-3 IOD compared to control (ACR-non-exposed group with normal diet). Simultaneous exposure of acrylamide and high-fat-intake did not significantly chang the Caspase-3 IOD compared to control too. The results above suggest that either acrylamide or high-fat-intake do not induce the expression of Caspase-3, as well as that of the combined.

**Table 3:** Expressions on protein level and activity of Caspase-3.

		Protein expression		Protein activity	
			
		*n*	IOD	*n*	Relative light units
NM	ACR 0 mg/kgbw•d	6	3127.83 ± 515.29	3	2354.23 ± 125.35
	ACR 20 mg/kgbw•d	6	2227.60 ± 524.83	3	2254.65 ± 235.18
	ACR 40 mg/kgbw•d	6	1661.40 ± 967.54	3	1998.54 ± 238.62
HF	ACR 0 mg/kgbw•d	6	4619.37 ± 104.48	3	2365.82 ± 135.26
	ACR 20 mg/kgbw•d	6	2809.08 ± 125.36	3	2553.42 ± 155.21
	ACR 40 mg/kgbw•d	6	2498.71 ± 525.74	3	2109.37 ± 366.81

*n* = number of mice used in experience, NM = normal diet block, HF = high-fat diet block. Compared with the control (ACR-non-exposed group with normal diet), no group had significant change on protein expression or activity of Caspase-3.

Because the cleavage of procaspase-3 to active Caspase-3 is essential to the activity of Caspase-3 (Pandey et al. 2000), we also investigated the activity of Caspase-3 in testis after detecting expression on protein level. Unilateral testes of three mice per treated group were homogenized, and then the supernatants were subjected to a Caspase-3 activity kit. Compared with the control, no group had significant change on activity of Caspase-3, which indicated that either acrylamide or high-fat-intake do not induce the activity of Caspase-3, as well as that of the combined.

## Discussion

The purpose of this present study is mainly to make sure if high-fat-intake has an altering effect on the reproductive toxicity of ACR through the mechanism of apoptosis, as well as the caspase-dependent pathway during the apoptotic process. Results coming from the present study are beneficial to determine the upper level of ACR in human diets and to determine the limited standard of ACR in relative foods such as fried potato chips, also to improve the techniques reducing ACR in questionably high-ACR foods.

We found the apoptosis induced by ACR in mice testes (*p* < 0.05); however, no significant apoptotic change was observed after the combined exposure of ACR and high-fat-intake (*p* > 0.05). As for the caspase-3, no significant protein expression and activity changes were observed in any treated group (*p* > 0.05). Moreover, no significant differences on ACR or GA concentration were observed between basic and high-fat diet group after ACR-exposure (*p* > 0.05). All the data suggest that high fat intake reverses the apoptosis induced by ACR; and, more possibly, apoptosis was induced by a caspase-3-independent mechanism. To our knowledge, this is the first finding of interaction between ACR and high-fat-intake. Based on the findings of the present study, we should reconsider the reproductive toxicity of ACR because of the high frequency of high-fat diet in daily life.

ACR toxicity appears to increase Leydig cell death and perturb gene expression levels, contributing to sperm defects and various abnormal histopathological lesions including apoptosis in rat testis (Yang et al. 2005). Our study demonstrated the apoptosis induced by ACR in mice testis. Acrylamide alone slightly increased apoptosis of bovine lens epithelial (BEL) cells; however, simultaneous exposure to ACR and staurosporine for 8 hours produced significantly less apoptosis than staurosporine alone, and pre-incubation with ACR followed by staurosporine for 8 and 24 hours markedly reduced apoptosis compared to staurosporine alone (Arocena 2006). ACR seems therefore to have a dual effect on BEL cell survival. Moreover, promoting effects of high-fat diets on chemical-induced mammary tumorigenesis, which is the result of abnormal apoptosis, was also reported (Reddy and Sugie 1988). The present study shows a consistent conclusion; simultaneous exposure to ACR and high-fat-intake produced significantly less apoptosis than ACR alone in mice testes.

Caspase-3, which is involved in the nuclear fragmentation at the final step of apoptosis, in particular, is associated with the execution of apoptosis (Shi 2002). In this study, DNA fragmentation and apoptotic body were induced in the ACR-treated group (*p* < 0.05) and was not induced in the group treated with ACR and high-fat together (*p* > 0.05), which was not consistent with the change of activity of caspase-3, as well as the capase-3 expression, so apoptosis was induced more possibly by a capase-3-independent pathway. More recently, studies show that mechanisms other than caspase-dependent apoptosis may be involved in the apoptotic process in the genesis of toxicity of ACR in SH-SY5Y cells (Tomoyuki and Hideki 2007).

Caspase-3-independent apoptosis, which were induced by radiofrequency fields in cortical neurons (Joubert et al. 2008), by ciglitazone in renal epithelial cells (Kwon et al. 2008), by oxidative stress in immortalized ganglion cell line (Li and Osborne 2008), by phenoxazine derivatives (2-amino-4,4 alpha-dihydro-4alpha-phenoxazine-3-oneand2-aminophe-noxazine-3-one) in human neuroblastoma cell line NB-1 cells (Shirato et al. 2007), and by porphyromonas gingivalis in fibroblast (Desta and Graves 2007), were reported more recently. So, in response to apoptotic stimuli, mitochondria can release caspase-independent cell death effectors. One of these effectors is apoptosis inducing factor (AIF), which could induce nuclear apoptosis in a variety of cell types. The effect was not inhibited by pharmacological caspase inhibitors such as zVAD, indicating that AIF can trigger nuclear apoptosis in a caspase-independent manner. AIF binds to DNA in a sequence-independent manner, which determines the entry of this complex into the nucleus. It recruits or activates an endonuclease to facilitate DNA fragmentation and chromatin condensation (Cregan et al. 2004). Calpain, a Ca^2+^-dependent intracellular cysteine protease in cell death, is also a caspase-independent cell death effector. Calpain is activated in various necrotic and an apoptotic condition, while caspase-3 is only activated in apoptosis. Caspases and calpains share several substrates, including PARP. During apoptosis, the 116 kDa PARP is degraded by caspase-3 to distinct 89 and 27 kDa fragments; however, PARP is cleaved by calpain at alternative sites to generate fragments from 70 to 40 kDa in size during necrosis (McGinnis et al. 1999). So calpain contributes to cell death and replaces caspase-3 to execute PARP activation in part (Qiao et al. 2007). In addition, Caspase-7 might substitute for capase-3 in most cell types and tissues (Wang 2000), since it is highly homologous to caspase-3 and has very similar substrate specificity. The present study failed to determine which other protein, except for caspase-3, is involved in calming down apoptosis. The role of proteins discussed above should be investigated in the future.

To conclude, ACR induces apoptosis in mice testis and high-fat-intake reverses the effects on ACR-induced apoptosis; and the apoptosis is induced more possibly through a caspase-3-independent pathway which is indicated by no activity of caspase-3 or lack of capase-3 expression.

## Abbreviations

ACR: Acrylamide
GA: glycidamide
IOD: integrated optical density
